# Fungistatic effect of sorbic acid on yeast cells via translational repression involving eIF2 α phosphorylation and formation of Ded1- and eIF2B-granules

**DOI:** 10.15698/mic2026.06.880

**Published:** 2026-06-17

**Authors:** Haruka Yoshiyama, Wataru Nomura, Shingo Izawa

**Affiliations:** 1 Laboratory of Microbiological Technology, Kyoto Institute of Technology, Matsugasaki, Kyoto 606-8585, Japan; 2 Division of Food Science and Biotechnology, Graduate School of Agriculture, Shinshu University, Nagano 399-4598, Japan

**Keywords:** sorbic acid, Ded1, eIF2B, stress granules, translational repression, fungistatic effect, *Saccharomyces cerevisiae*

## Abstract

Sorbic acid is a lipophilic weak acid with fungistatic activity, and it has been widely used as a food preservative, along with its potassium and calcium salts. Although the fungistatic effect of sorbic acid is thought to be primarily due to acidification within fungal cells, the detailed fungistatic mechanism remains unclear. We investigated the effects of sorbic acid on yeast translation in *Saccharomyces cerevisiae*. At sublethal concentrations (2–4 mM), sorbic acid quickly repressed translation. Conversely, removal of sorbic acid restored translation activity, indicating that the sorbic acid-induced translational repression is reversible. Pronounced translational repression induced by various stress conditions or nutrient starvation is often accompanied by eIF2 α phosphorylation, eIF2B-body and stress granule (SG) formation, and the sequestration of Ded1 (which plays a role in translation initiation as a DEAD-box RNA helicase) into SGs. We found that sorbic acid stress also induces eIF2 α phosphorylation and the sequestration of Ded1 into SGs. In contrast, sorbic acid stress induced the formation of not eIF2B bodies but eIF2B granules, which colocalized with SGs. These results suggest that the functional arrest of translation-related factors, including eIF2 α , eIF2B, and Ded1, correlates strongly with the translational repression in the presence of sorbic acid. Notably, Gcn2 deficiency delayed translational repression and SG formation, and significantly suppressed eIF2B granule formation, suggesting the involvement of Gcn2 in these stress responses during sorbic acid stress. Our findings provide new insights into the physiological effects of sorbic acid on yeast cells, specifically regarding the regulation of translation-related factors.

## INTRODUCTION

Sorbic acid is a weak lipophilic acid that exhibits fungistatic activity and has been widely used as a preservative primarily to prevent mold growth in foods and beverages 1, 2. The fungistatic effect of sorbic acid is thought to be mainly due to acidification within fungal cells 3–5. A correlation between sorbic acid-induced decreases in intracellular pH (pHi) and growth inhibition has been confirmed in the budding yeast *Saccharomyces cerevisiae* 6. It is noteworthy that sorbic acid exhibits fungistatic effects against *S. cerevisiae* at concentrations significantly lower than those required for acetic acid; however, it displays a more modest ability to reduce pHi of yeast cells than acetic acid 6–10. In contrast, sorbic acid interferes with the mitochondrial membrane potential and raises intracellular reactive oxygen species (ROS) levels 7, 11. Therefore, yeast cells undergoing respiration are more sensitive to sorbic acid than yeast cells undergoing fermentation 11, 12. A comprehensive screening of genes associated with sorbic acid susceptibility and resistance also supports the involvement of intracellular acidification and increased ROS levels in the antifungal activity 13–15.

Genome-wide analyses of yeast responses to sorbic acid have revealed altered transcription patterns mediated by the stress-responsive transcription factors War1 and Msn2/Msn4 14, 16–18. Although these analyses provide valuable insights into how yeast responds to sorbic acid stress, transcript expression levels do not necessarily reflect the levels of translated products in eukaryotes 19, 20. Indeed, among all genes whose transcript levels increased due to 0.9 mM sorbic acid, only Hsp26 displayed elevated protein levels 16. Currently, information on the effects of sorbic acid stress on translation activity is sparse.

Certain types of stress inhibit translation activity and arrest yeast growth even under nutrient-rich conditions. Heat shock and severe ethanol stress are well known to repress translation activity in yeast cells during the exponential growth phase (log phase) 20–24, and fermentation inhibitors, including vanillin, furfural, and xylene also repress translation 25–27. Pronounced translational repression is often accompanied by stress granule (SG) formation. Indeed, SG formation has been observed under the aforementioned stress conditions and during glucose depletion 21, 25–31. SGs contain non-translated mRNAs and various proteins, including translation-related factors 31. SG formation is involved in the inactivation and protection of translation-related factors and non-translating mRNAs under stress conditions, and it contributes to the rapid resumption of translation after stress elimination 31–33.

Three major changes in translation-related factors during translational repression have been reported. As one of the well-studied phenomena, phosphorylation of eIF2 α by the protein kinase Gcn2 is known to block the regeneration of eIF2-GTP from eIF2-GDP by eIF2B, resulting in reduced levels of available ternary complex (eIF2-GTP- ${\text{tRNA}}_{i}^{\mathrm{Met}}$ ) 34–36. In *S. cerevisiae*, nitrogen starvation stress is well known to enhance the levels of phosphorylated eIF2 α 37. The second change is the formation of eIF2B bodies in yeast cells. eIF2B is a heterodecamer composed of five subunits (two copies of each subunit): Gcn3 (eIF2B- α ), Gcd7 (eIF2B- β ), Gcd1 (eIF2B- γ ), Gcd2 (eIF2B- δ ), and Gcd6 (eIF2B- ɛ ) 38, 39. eIF2B often forms filamentous structures via self-polymerization, and these filaments are called eIF2B bodies. Although eIF2B body formation is observed even in log-phase cells 40–42, it is further activated under conditions of translational repression, including glucose starvation 39, 43–45. A model has been proposed in which eIF2B activity is inhibited by the filamentous structure partially blocking the catalytic site of Gcd6, thereby contributing to translational repression and to protection of eIF2B under stress conditions 39.

The third phenomenon is the sequestration of Ded1, an essential DEAD-box RNA helicase, in SGs. During translation initiation, Ded1 unwinds the secondary structure in 5’-untraslated region (UTR) of mRNA, enabling scanning by 43S pre-initiation complexes to identify start codons 46. Ded1 function is thus crucial for translation of mRNAs containing long structured 5’-UTRs 47, 48. Under several stress conditions including glucose depletion and heat shock, Ded1 is inactivated through dissociation from mRNA, formation of granules, and sequestration into SGs 22–24, 49. The existence of functional arrests for multiple translation-related factors, including eIF2 α , eIF2B, and Ded1, along with their selective utilization, might be advantageous, allowing yeast cells to respond appropriately to diverse stresses.

Beyond intracellular acidification and disruption of mitochondrial membrane potential, the specific effects of sorbic acid on yeast cells, including its impact on translation activity, remain unclear, and the mechanism underlying its fungistatic action has yet to be fully elucidated. In this study, we investigated whether translational repression was involved in the fungistatic effects of sorbic acid on *S. cerevisiae*. We found that 4 mM sorbic acid represses translation with eIF2 α phosphorylation, eIF2B granule but not eIF2B body formation, and Ded1 sequestration in SGs. These findings provide novel insights into the physiological effects of sorbic acid on yeast cells.

## RESULTS

### Sorbic acid causes translational repression

**Figure 1:**
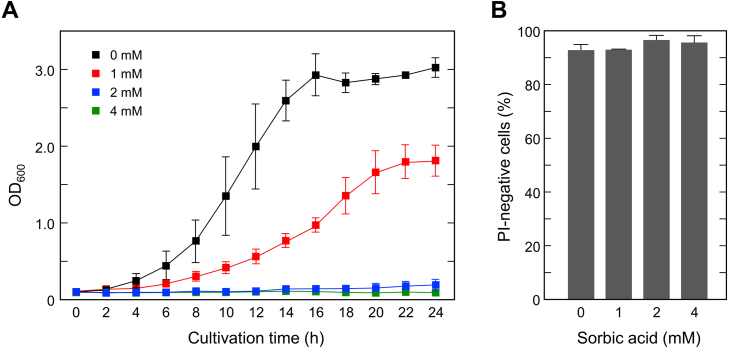
Inhibition of cell proliferation by sorbic acid without rapid cell death. Yeast cells were cultured in SD medium containing 1, 2, or 4 mM sorbic acid at 28 ${}^{\circ }$ C. **(A)** Cell growth was monitored by measuring optical density at 600 nm (OD ${}_{600}$ ). **(B)** The cell death rate after treatment with sorbic acid for 24 h was measured using propidium iodide (PI) staining.

To confirm the fungistatic effects of sorbic acid, yeast cells were cultured at 28 ${}^{\circ }$ C in synthetic defined (SD) medium containing sorbic acid. Yeast cells could proliferate in the presence of 1 mM sorbic acid, but their growth was strongly inhibited at 2 mM or higher (Figure 1**A**). Propidium iodide (PI) staining revealed that the cell death rate remained low (less than 10%) 24 h after sorbic acid treatment (Figure 1**B**). These results indicate that sorbic acid at concentrations of 2–4 mM inhibits yeast cell proliferation, but does not cause lethal stress.

**Figure 2:**
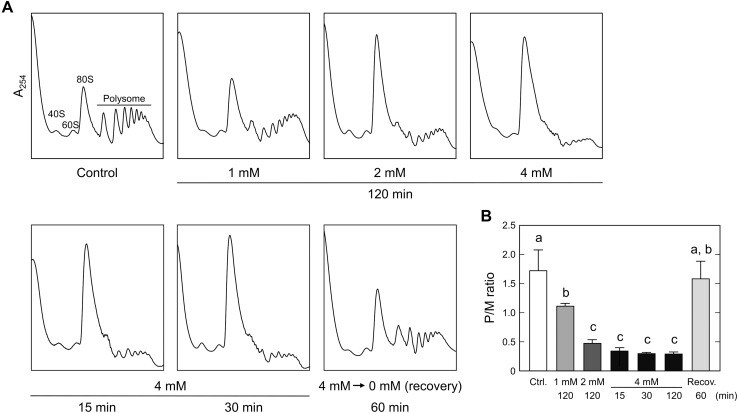
Sorbic acid causes translational repression in yeast cells. **(A)** Polysome profiles were determined in yeast cells under sorbic acid stress. Yeast cells were treated with 1, 2, or 4 mM sorbic acid at 28 ${}^{\circ }$ C. In the recovery experiment, cells were treated with 4 mM sorbic acid for 120 min, then incubated for 60 min in fresh SD medium without sorbic acid. **(B)** Polysome/monosome (P/M) ratios under each condition were calculated (mean ± S.D., *n* = 3). Different letters indicate statistically significant differences (*p* < 0.05, ANOVA with post-hoc Tukey’s test).

We performed polysome analysis to evaluate the effect of sorbic acid on yeast translation activity (Figure 2). The polysome/monosome (P/M) ratio was calculated as an indicator of translation activity 50. Although 1 mM sorbic acid exerted only a slight effect on the P/M ratio, 4 mM sorbic acid caused an increase in the 80S monosome peak and a significant decrease in the polysome peaks, demonstrating that 4 mM sorbic acid markedly repressed translation. Sorbic acid (4 mM)-induced translational repression was observed within 15 min, demonstrating rapid efficacy. Translation activity largely recovered within 60 min after sorbic acid removal by medium exchange, indicating that sorbic acid-induced translational repression is reversible.

### Sorbic acid causes eIF2 α phosphorylation and formation of eIF2B granules

**Figure 3:**
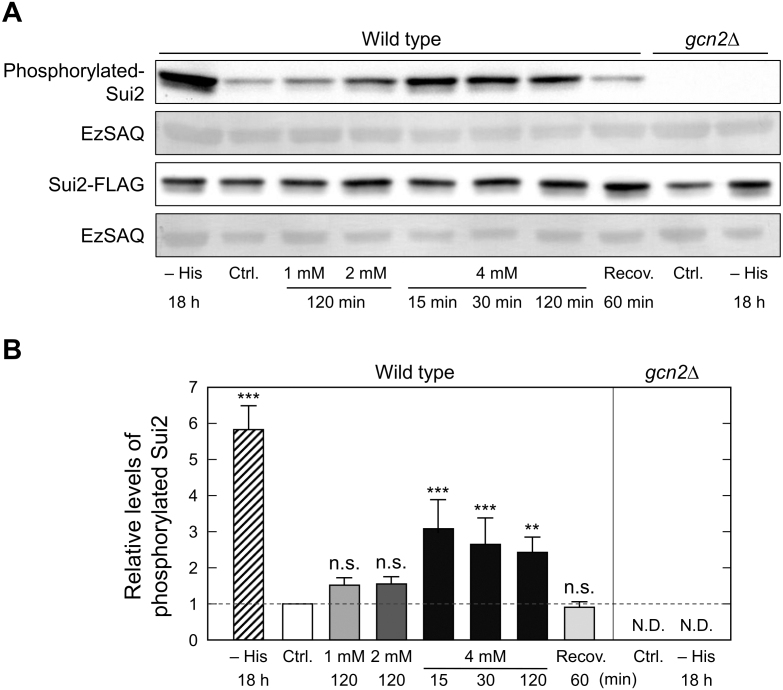
Sorbic acid causes eIF2 α phosphorylation. **(A)** Phosphorylated eIF2 α (Sui2) levels were assayed via western blotting. Yeast cells (wild-type and *gcn2* Δ ) expressing Sui2-FLAG were treated with 1, 2, or 4 mM sorbic acid at 28 ${}^{\circ }$ C. In the recovery experiment, cells were treated with 4 mM sorbic acid for 120 min, then incubated for 60 min in fresh SD medium without sorbic acid. Cells were also exposed to amino acid starvation (– His) for 18 h. **(B)** Phosphorylated eIF2 α (Sui2-P) levels were quantified by normalizing Sui2-P to Sui2-FLAG band intensities, after normalizing the intensity of each lane using EzSAQ staining (mean ± S.D., *n* = 3). The Sui2-P level in cells not subjected to stress treatment (Ctrl.) was set to a relative value of 1. Statistical significance compared with the Ctrl. group of wild-type cells was evaluated using Dunnett’s post-hoc test. ^⁎⁎⁎^*p* < 0.001, ^⁎⁎^*p* < 0.01, n.s., statistically not significant. N.D., not detected.

To elucidate the mechanism underlying translational repression, we examined whether sorbic acid causes eIF2 α phosphorylation, which is known to suppress translation by reducing the levels of the ternary complex needed for initiation 34–36. We confirmed that, as previously reported, eIF2 α phosphorylation is enhanced under amino acid starvation stress (–His), which also induces translational repression 24, 37 (Figure 3). This phosphorylation was not detected in *gcn2* Δ cells, as expected, given that eIF2 α phosphorylation is Gcn2-dependent 34. In the presence of sorbic acid, a concentration-dependent increase in eIF2 α phosphorylation levels was observed, with 4 mM sorbic acid inducing significant eIF2 α phosphorylation within 15 min. The elimination of 4 mM sorbic acid reduced eIF2 α phosphorylation levels. These results suggest that eIF2 α phosphorylation contributes to translational repression caused by sorbic acid.

**Figure 4:**
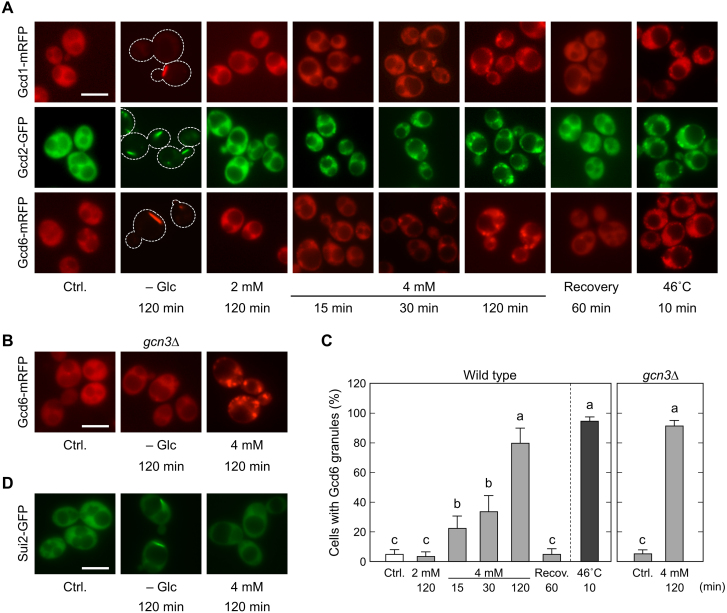
Sorbic acid causes the formation of eIF2B granules but not eIF2B bodies. **(A, B)** Intracellular localizations of eIF2B components (Gcd1, Gcd2, and Gcd6) were examined in cells (wild-type and *gcn3* Δ ). Yeast cells were treated with sorbic acid stress (2 and 4 mM) or glucose depletion (– Glc) at 28 ${}^{\circ }$ C. In the recovery experiment, cells were treated with 4 mM sorbic acid for 120 min, then incubated for 60 min in fresh SD medium without sorbic acid. Cells were also exposed to heat shock at 46 ${}^{\circ }$ C for 10 min. **(C)** The percentage of cells containing eIF2B granules was measured using Gcd6-mRFP. Different letters indicate statistically significant differences (*p* < 0.05, ANOVA with post-hoc Tukey’s test). **(D)** Wild-type cells expressing Sui2-GFP were treated with glucose depletion or 4 mM sorbic acid stress for 120 min. Scale bar, 5 μ m.

We then examined whether sorbic acid induces eIF2B body formation. We confirmed filamentous eIF2B body formation under glucose depletion, as previously reported 44, 45, with eIF2B components, Gcd1 (eIF2B- γ ), Gcd2 (eIF2B- δ ), and Gcd6 (eIF2B- ɛ ) (Figure 4**A**). In contrast, 4 mM sorbic acid and severe heat shock at 46 ${}^{\circ }$ C induced the formation of non-filamentous eIF2B granules but not eIF2B bodies (Figures 4**A** and 4**C**). Formation of eIF2B granules was reversible upon sorbic acid removal, and eIF2B granule formation was observed even in *gcn3* Δ cells, in which filamentous eIF2B bodies cannot form 51 (Figures 4**B** and 4**C**). These findings suggest that sorbic acid inactivates eIF2B by forming eIF2B granules that are distinct from eIF2B bodies.

Although Sui2 is known to form filamentous structures and colocalize with eIF2B bodies upon glucose depletion 40, 41, 45, Sui2-GFP neither formed foci nor colocalized with eIF2B granules under sorbic acid stress (Figure 4**D**).

### Sorbic acid causes Ded1 sequestration in stress granules

**Figure 5:**
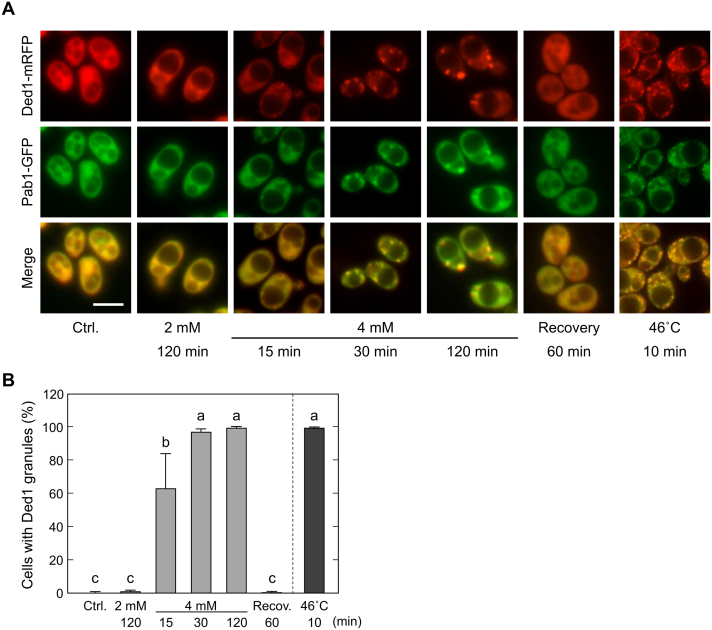
Ded1 is sequestered into stress granules under sorbic acid stress. **(A)** Wild-type cells expressing Ded1-mRFP and Pab1-GFP (an SG marker) were treated with sorbic acid stress or heat shock. Scale bar, 5 μ m. **(B)** The percentage of cells containing Ded1 foci was measured. Different letters indicate statistically significant differences (*p* < 0.05, ANOVA with post-hoc Tukey’s test).

Next, we examined whether sorbic acid induces Ded1 granule formation. Treatment with 4 mM sorbic acid caused Ded1 granule formation, as did heat shock and severe ethanol stress 23, 24 (Figure 5). These Ded1 granules co-localized with Pab1-GFP, an SG marker 24, in the cytoplasm under sorbic acid stress, indicating Ded1 sequestration in SGs. The Ded1 granules and SGs formed by 4 mM sorbic acid mostly disappeared within 60 min of sorbic acid removal.

**Figure 6:**
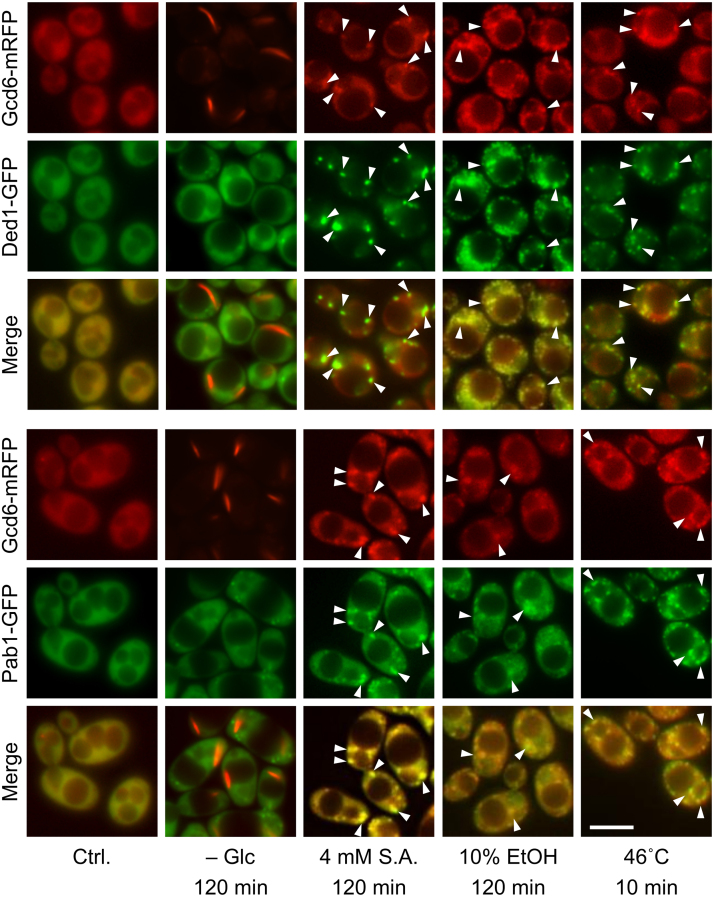
eIF2B also co-localizes with stress granules under sorbic acid stress. Co-localization of Ded1-GFP and Gcd6-mRFP, as well as Pab1-GFP and Gcd6-mRFP, was examined under stress conditions. Wild-type cells were exposed to the indicated stress conditions. White arrowheads indicate colocalization sites for Gcd6 and Ded1 or Gcd6 and Pab1. S.A., sorbic acid. Scale bar, 5 μ m.

We examined whether eIF2B granules also co-localize with SGs under sorbic acid stress. As shown in Figure 6, Gcd6 co-localized with Ded1 and Pab1 under 4 mM sorbic acid stress as well as under heat shock (46 ${}^{\circ }$ C) and severe ethanol stress (10% *v/v*). These results indicate that sorbic acid induces the localization of the translation-related factors, Ded1 and eIF2B, to SGs. Furthermore, it was revealed that heat shock and severe ethanol stress also induce the formation of eIF2B granules and their localization to SGs.

### Translational repression and formation of eIF2B granules in *gcn2* Δ cells

**Figure 7:**
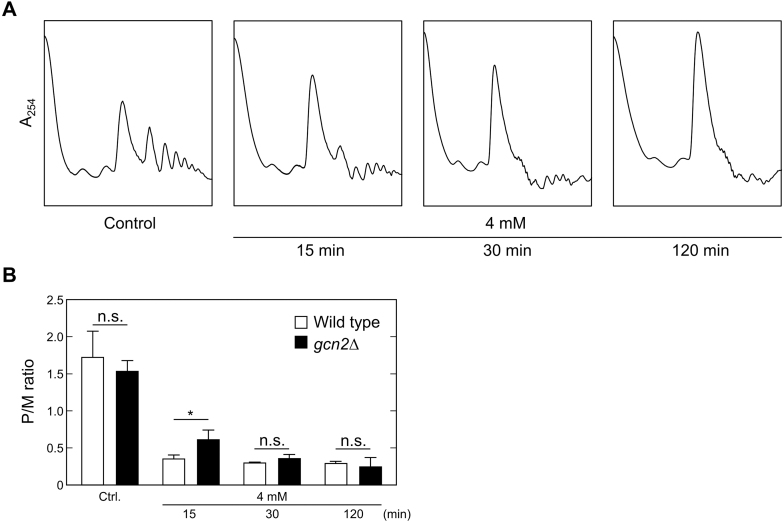
Translational repression in *gcn2* Δ cells under sorbic acid stress. *gcn2* Δ cells were exposed to 4 mM sorbic acid stress for the indicated time. **(A)** Polysome profiles were determined in *gcn2* Δ cells under sorbic acid stress. **(B)** P/M ratios under each condition were calculated (mean ± S.D., *n* = 3). ^⁎^*p* < 0.05, n.s., statistically not significant (Tukey’s adjustment with the emmeans R package). The data for wild-type cells were taken from Figure 2.

**Figure 8:**
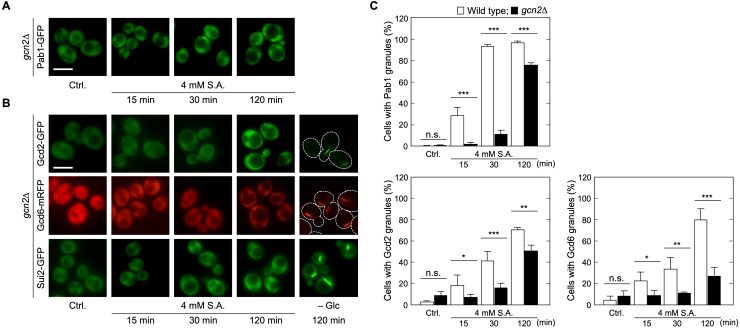
Granule formation of eIF2B and Pab1 in *gcn2* Δ cells under sorbic acid stress. **(A, B)** Intracellular localizations of Pab1, eIF2B components (Gcd2 and Gcd6), and Sui2 were examined in *gcn2* Δ cells under indicated conditions. S.A., sorbic acid. Scale bar, 5 μ m. **(C)** The percentage of cells containing granules of each component was measured. ^⁎⁎⁎^*p* < 0.001, ^⁎⁎^*p* < 0.01, ^⁎^*p* < 0.05, n.s., statistically not significant (Tukey’s adjustment with the emmeans R package).

Gcn2 inhibits translation initiation not only through eIF2 α phosphorylation but also via phosphorylation of eIF2 β and Gcn20 52. While *gcn2* Δ cells exhibit delayed translational repression under amino acid starvation and rapamycin treatment 53–56, Gcn2 deficiency has been reported not to affect translational repression and eIF2B body formation induced by glucose depletion 38, 45, 57. To assess the role of Gcn2 in the response to sorbic acid stress, we performed polysome analysis and examined the formation of SGs and eIF2B granules in *gcn2* Δ cells. Polysome analysis revealed that the P/M ratio was higher in *gcn2* Δ cells than in wild-type cells at 15 min after the initiation of sorbic acid treatment (Figure 7). A similar trend of higher P/M ratio in *gcn2* Δ cells was observed at 30 min. Consistent with this, the formation of SGs also tended to be delayed in *gcn2* Δ cells compared to wild-type cells (Figures 8**A** and 8**C**). These results suggest that sorbic acid-dependent translational repression is delayed in the absence of Gcn2.

Furthermore, the loss of Gcn2 had a profound effect on eIF2B granule formation; the frequency of granule formation under sorbic acid stress was significantly lower in *gcn2* Δ cells than in wild-type cells (Figures 8**B** and 8**C**). Consistent with previous findings 45, glucose starvation induced filamentous eIF2B bodies in *gcn2* Δ cells, similarly to wild-type cells (Figure 8**B**).

## DISCUSSION

We demonstrated that sorbic acid represses translation activity via eIF2 α phosphorylation and granule formation by Ded1 and eIF2B. Translational repression by sorbic acid appears to contribute to its fungistatic effects in yeast cells; inhibiting *de novo* protein synthesis suppresses yeast proliferation, preventing food spoilage.

Although eIF2 α phosphorylation is a well-known response to various environmental stresses, such as nutrient limitation, rapamycin, methylglyoxal, and iron deficiency 54, 58–60, our study provides the first direct evidence that food additives and preservatives can trigger this conserved pathway. Consequently, whether other preservatives, like benzoic acid or natamycin, exert similar effects on eIF2 α is a compelling question for future research. Although the levels of sorbic acid-induced eIF2 α phosphorylation decreased over time, the translational repression persisted, likely due to factors independent of eIF2 α phosphorylation, such as the dysfunction of eIF2B and Ded1.

Recent studies have provided significant insights into the activation mechanisms of Gcn2. Under non-stressed conditions, Gcn2 exists as a dimer associated with the free 60S ribosomal subunit; however, it is reportedly activated by ribosome collisions or uncharged tRNAs under stress conditions 56, 61. Therefore, a crucial next step is to investigate whether sorbic acid causes ribosome stalling or interferes with tRNA charging to drive Gcn2-dependent stress responses.

To date, reports of eIF2B granule formation are scarce, with most literature focusing on filamentous eIF2B bodies. Regarding the spatial organization of eIF2B, our results suggest that eIF2B granules and eIF2B bodies are distinct entities. A defining difference lies in their relationship with SGs; whereas eIF2B bodies show minimal colocalization with SGs 44, our findings indicate that eIF2B granules are integral components of, or closely associated with, sorbic acid-induced SGs. Notably, *gcn3* Δ cells retained the ability to form eIF2B granules despite their reported incapacity to form filamentous eIF2B bodies 51. Furthermore, since eIF2B granules colocalized with SGs, they may represent disordered assemblies rather than the structured, decamer-based assemblies characteristic of eIF2B bodies 39.

Since SG composition is stress-type dependent 62–64, targeting eIF2B to sorbic acid-induced SGs likely represents a coordinated response for efficient translational control and the protection of eIF2B. While eIF2B localizes to SGs along with other translation-related factors under heat shock, severe ethanol stress, and sorbic acid stress, its behavior under glucose depletion is distinct; in this condition, eIF2B forms filamentous bodies independent of SGs 44. Morphological differences in eIF2B under glucose depletion and sorbic acid stress suggest that these stresses possess distinct properties. Furthermore, the absence of Ded1 sequestration into SGs under amino acid starvation 24 indicates that sorbic acid stress is not equivalent to amino acid starvation stress.

In response to sorbic acid stress, we observed a delay in both translational repression and SG formation in *gcn2* Δ cells. These results parallel earlier reports 53–56 showing that translational repression, triggered by amino acid starvation or rapamycin treatment, is delayed by Gcn2 dysfunction. Furthermore, we found that Gcn2 plays a differential role in the formation of eIF2B granules and eIF2B bodies. The formation rate of eIF2B granules significantly decreased in *gcn2* Δ cells under sorbic acid stress, whereas Gcn2 was dispensable for eIF2B body formation (Figure 7**D**) 45. This finding suggests that Gcn2 activation and the subsequent phosphorylation of its targets, such as eIF2 α , may trigger the formation of eIF2B granules under sorbic acid stress. Although the precise mechanism by which Gcn2 facilitates eIF2B granule formation under sorbic acid stress remains to be elucidated, this study highlights the distinctive characteristics of eIF2B granules and eIF2B bodies.

In conclusion, this study revealed that sorbic acid represses yeast translation activity, causing eIF2 α phosphorylation, Ded1 sequestration into SGs, and the formation of eIF2B granules. These eIF2B granules, also induced by heat shock and severe ethanol stress, possess characteristics distinct from previously reported eIF2B bodies and co-localize with Ded1 and SGs. Furthermore, Gcn2 deficiency delays translational repression and significantly suppresses the formation of eIF2B granules, suggesting its crucial role in these stress-responsive mechanisms during sorbic acid stress.

## MATERIALS AND METHODS

### Yeast strains and stress treatment

Parental wild-type *S. cerevisiae* BY4742 strain (*MAT* α *ura3* Δ *0 his3* Δ *1 leu2* Δ *0 lys2* Δ *0*) and its isogenic knockout mutants (*gcn2* Δ and *gcn3* Δ ) were purchased from Open Biosystems (Huntsville, AL, USA). C-terminal tagging of Gcd2 with GFP was performed as described by Longtine *et al*. 65 using pFA6a-*GFP*-His3MX6 and *GCD2*-FA-F1/R1 primer sets (Table 1). Yeast cells were cultured in SD medium (2% glucose, 0.67% yeast nitrogen base without amino acids, 20 mg/L uracil, 30 mg/L l-lysine HCl, 100 mg/L l-leucine, and 20 mg/L l-histidine HCl, pH 5.6) with reciprocal shaking (120 rpm) at 28 ${}^{\circ }$ C, and exponentially growing cells were harvested at an OD ${}_{600}$ of 0.5. Harvested cells were transferred to fresh SD medium containing sorbic acid or ethanol for stress treatment. Heat shock treatment was performed by transferring yeast cells to SD medium pre-warmed to 46 ${}^{\circ }$ C and incubated at 46 ${}^{\circ }$ C for 10 min.

**Table 1:** List of primers used to construct the strains and plasmids .

Name	Sequence
*GCD1*-F1	5 ${}^{\prime }$ -CTATAGGGCGAATTGGAGCTCCCTAAGAAAGCAACAA TTTGAACT-3 ${}^{\prime }$

*GCD1*-R1	5 ${}^{\prime }$ -CGTCCTCGGAGGAGGCTCGAGAACGCTCAAATAATCC GTCATCTT-3 ${}^{\prime }$

*GCD6*-F1	5 ${}^{\prime }$ -CTATAGGGCGAATTGGAGCTCTAGAGAGAAGACTTCC ATTCAG-3 ${}^{\prime }$

*GCD6*-R1	5 ${}^{\prime }$ -CGTCCTCGGAGGAGGCTCGAGATTCCTCTTCTGAGGAA GATTCTT-3 ${}^{\prime }$

*GCD2*-FA-F1	5 ${}^{\prime }$ -TTTAAGAGAGTACAAAGGTTCCGCACGGATCCCCGGGT TAATTAA-3 ${}^{\prime }$

*GCD2*-FA-R1	5 ${}^{\prime }$ -CCATCTTGCCTCCTGCTAATGTGGCGAATTCGAGCTCGT TTAAAC-3 ${}^{\prime }$

### Plasmids

To construct integrative plasmids for the expression of mRFP-tagged proteins, the 3’-terminal regions (excluding the stop codon) of the *GCD1* and *GCD6* open reading frames were cloned into YIp-*DED1-*mRFP 24. These 3’-terminal regions were amplified by PCR using F1/R1 primer sets (Table 1) and genomic DNA of BY4742 as template. The resulting plasmids, YIp-*GCD1-mRFP* and YIp-*GCD6-mRFP*, were linearized with *Cla*I and *Eco*RI, respectively, and introduced into the corresponding chromosomal loci. This allowed the expression of each gene to be driven by its native promoter. The genomes of the transformants contained a single copy of the mRFP-tagged gene, with no untagged genes present. YIp-*PAB1-GFP*, YIp-*SUI2-GFP*, YIp-*SUI2-FLAG*, YIp-*DED1-GFP*, and YIp-*DED1-mRFP* were described previously 24, 62.

### Polysome analysis

Polysome analysis was conducted by the method of Inada and Aiba 66 using a gradient master and fractionator (107–201M and 152–002; BioComp Instruments, Fredericton, NB, Canada). The polysome/monosome (P/M) ratio was calculated (area under the polysomal ribosome peaks/area under the 80S monosome peak) as an indicator of translation activity 50.

### Fluorescent microscopic analysis and western blotting

For the fluorescent microscopic analysis, yeast cells were observed immediately after stress treatment without fixation, using an IX83 microscope system (Olympus, Tokyo, Japan) with a Digital CMOS camera (C11440-22CU, Hamamatsu Photonics K.K., Shizuoka, Japan), an acquisition software (OLYMPUS cellSens Dimension 1.18), and a personal computer (Precision T3610, Dell Technologies Inc., TX, USA). The cell death rate was assessed using PI staining 67. To obtain quantitative data on the formation of granules, over 100 living cells were examined under each condition, and the experiments repeated three times (more than 300 cells in total).

Anti-FLAG (F1804; Sigma-Aldrich, MO, USA) and anti-mouse IgG, HRP-linked (7076S; Cell Signaling Technology, MA, USA) antibodies were used for western blotting to detect the FLAG-tagged proteins. To monitor levels of phosphorylated eIF2 α , anti-phospho-eIF2S1 (Ser52) polyclonal antibody (#44–728G; Thermo Fisher Scientific, MA, USA) and anti-rabbit IgG antibody, HRP-linked (406401; BioLegend, CA, USA) were used. The bands on the western blots were quantified using ImageJ and normalized to total protein levels. To confirm equal loading and the transfer of all proteins, the membranes were stained with EzStainAQua MEM (EzSAQ) (WSE-7160; ATTO Corporation, Tokyo, Japan).

### Statistical analysis

Statistical significance was assessed using the Dunnett’s (Figure 3) or Tukey’s (Figures 2, 4, and 5) post-hoc test with RStudio (https://posit.co/products/open-source/rstudio/). For Figures 7 and 8, two-way ANOVA was performed, followed by pairwise comparisons of strains within each condition using the emmeans R package with Tukey’s adjustment. Data are presented as means ± standard deviations (S.D.).

## DATA AVAILABILITY

All data discussed in this article is available upon request.

## AUTHOR CONTRIBUTIONS

H.Y.: conceptualization; investigation; writing-original draft preparation. W.N.: investigation; writing-reviewing and editing. S.I.: conceptualization; project administration; funding acquisition; writing-reviewing and editing.

## CONFLICT OF INTEREST

No potential conflicts of interest were reported by the authors.

## ABBREVIATIONS

PI – propidium iodide

ROS – reactive oxygen species

SD medium – synthetic defined medium

SGs – stress granules

UTR – untranslated region
